# Ligand-mediated changes in conformational dynamics of NpmA: implications for ribosomal interactions

**DOI:** 10.1038/srep37061

**Published:** 2016-11-15

**Authors:** Nilofer Husain, Nikhil Kumar Tulsian, Wang Loo Chien, Sushant Suresh, Ganesh Srinivasan Anand, J. Sivaraman

**Affiliations:** 1 Department of Biological Sciences, 14 Science Drive 4, National University of Singapore, 117543 Singapore; 2 Manipal College of Pharmaceutical Sciences, Manipal, 576104 India

## Abstract

Aminoglycosides are broad-spectrum antibiotics that bind to the 30S ribosomal subunit (30S) of bacteria and disrupt protein translation. NpmA, a structurally well-characterized methyltransferase identified in an *E. coli* clinical isolate, catalyzes methylation of 30S at A1408 of the 16S rRNA and confers aminoglycoside resistance. Using sucrose cushion centrifugation and isothermal titration calorimetry, we first confirmed the binding between NpmA and 30S. Next, we performed amide Hydrogen/Deuterium Exchange Mass Spectrometry (HDXMS) of *apo* NpmA and in the presence and absence of SAM/SAH. We observed that ligand binding resulted in time-dependent differences in deuterium exchange not only at the ligand-binding pocket (D25–D55 and A86–E112) but also in distal regions (F62-F82 and Y113-S144) of NpmA. These results provide insights into methylation group donor cofactor-mediated allostery in NpmA in the ligand-bound states, which could not be observed in the static endpoint crystal structures. We predict that the two distal sites in NpmA form part of the allosteric sites that importantly are part of the main 16S rRNA binding interface. Thus HDXMS helped uncover allosteric communication relays that couple SAM/SAH binding sites with the ribosome-binding site. This highlights how HDXMS together with X-ray crystallography can provide important allosteric insights in protein-ligand complexes.

Serious infectious diseases caused by Gram-negative and Gram-positive bacteria are commonly treated with aminoglycosides^1^. These broad-spectrum antibiotics bind to the A-site of the 30S subunit of the bacterial ribosome, causing increases in translation errors and, consequently, a decrease in the production of functional proteins^2^. Various crystal structures showing the interactions between aminoglycosides and the 30S ribosomal subunit and RNA oligonucleotides that mimic the A-site of the 30S ribosomal subunit have been reported^3^,^4^,^5^,^6^. Overall, these structures revealed that at least eight contacts between the RNA and the tested aminoglycosides are conserved. Ring I of the aminoglycoside intercalates into the A-site helix and forms a pseudo-base pair with residue A1408, and it is believed that this interaction is responsible for the elevation in reading errors caused by the binding of aminoglycosides to the 30S ribosomal subunit^4^.

Excessive use of aminoglycoside antibiotics, however, has resulted in selection and the rampant progression and propagation of bacterial strains with resistance to these antibiotics. Specifically, methylation of the target site in bacteria hinders the binding of aminoglycosides to the ribosome. This methylation is carried out by methyltransferases (MTases) belonging to the Arm (aminoglycoside resistance MTase) and Kam (kanamycin-apramycin MTase) families^7^, which methylate at G1405 and A1408, respectively, on the 16S rRNA of the 30S ribosomal subunit. Given that ring I of aminoglycoside makes hydrogen bonding contacts with A1408 of the 16S rRNA, methylation at this site prevents ring I intercalation and hinders the action of the antibiotic.

The novel plasmid-mediated 16S rRNA m^1^A1408 MTase (NpmA) belongs to the Kam family and was identified in an *E. coli* clinical isolate^8^. NpmA methylates the fully assembled 30S ribosomal subunit at the N^1^ position of A1408^8^, and utilizes S-adenosyl-methionine (SAM) as a methyl group donor, giving rise to the post methylation endproduct, S-adenosyl-homocysteine (SAH). The structures of the *apo* and ligand-bound NpmA (NpmA-SAM and NpmA-SAH) were solved by our group^9^ and others^10^,^11^. NpmA adopts a Rossmann fold with a seven-stranded β sheet and belongs to the Class I MTases. We identified three key residues, D30, W107 and W197, which are required for the methylation function of NpmA and studied the ribosome interaction with NpmA by RNA foot printing and computational docking.

As a continuation of our efforts to understand the mechanism of NpmA-mediated antibiotic resistance, we purified the 30S ribosomal subunit from *E. coli*^12^ and performed sucrose cushion centrifugation and isothermal titration calorimetry (ITC) experiments to confirm the binding between NpmA and the 30S ribosomal subunit. We also set out to identify SAM/SAH-mediated allostery in NpmA that might enable a description of the cross-talk between 30S and SAM/SAH binding sites on NpmA. We therefore carried out amide hydrogen/deuterium exchange mass spectrometry (HDXMS) experiments with NpmA in the SAM- and SAH-bound states. While crystal structures have provided at high resolution, the SAM and SAH binding sites, these only represent snapshots of stabilized conformational end states. We wanted to capture the conformational dynamics of the protein in solution and determine insights into the allosteric effects of SAM and SAH interactions. In order to complement our structural studies by X-ray crystallography, we have applied HDXMS^13^ which offers insights into the allosteric effects of ligand binding^14^. By mapping regions of NpmA that show changes in HDXMS, outside the binding site, we have identified distal hotspots sites, which are the allosteric hotspots. Identification of allosteric hotspots with implications for ribosome binding offers opportunities to re-establish aminoglycoside sensitivity in resistant bacteria.

## Materials and Methods

### Expression and Purification of the Ribosome

The ribosomes used in this study have a tetra-(His)_6_ tag and were expressed and purified from *E. coli* strain JE28. This strain was obtained from Dr. Suparna Sanyal from Uppsala University, Sweden. The expression and purification was carried out as described earlier^12^. Briefly, 1 L of LB medium containing 50 μg/ml kanamycin was inoculated with 4 ml of overnight culture, which had been cultured at 37 °C until an A_600_ nm of 1.0 and then cooled to 4 °C to accumulate the “run-off” ribosomes. The 1 L culture was then centrifuged at 3500× g for 10 min at 4 °C to harvest the cells. The pellet was stored at −80 °C or re-suspended in lysis buffer comprising 20 mM Tris–HCl pH 7.6, 10 mM MgCl_2_, 150 mM KCl, 30 mM NH_4_Cl, protease inhibitor (Sigma-Aldrich), lysozyme (0.5 mg/ml) and DNAse I (10 μg/ml) for purification. After sonication, the cell lysate was centrifuged twice at 20,000 × g for 10 min at 4 °C.

### Affinity Purification

The lysate was injected into a HiTrap^TM^ Chelating HP column (5 ml; GE Healthcare) connected to an AKTA prime chromatography system (GE Healthcare). The system was equilibrated with lysis buffer and the column washed until a baseline level was reached. The column was then washed with wash buffer (20 mM Tris–HCl pH 7.6, 10 mM MgCl_2_, 150 mM KCl, 30 mM NH_4_Cl and 5 mM imidazole) and the 70S ribosome was eluted using an elution buffer containing a high concentration of imidazole (20 mM Tris–HCl pH 7.6, 10 mM MgCl_2_, 150 mM KCl, 30 mM NH_4_Cl and 150 mM imidazole).

To separate the ribosomal subunits, the 70S ribosome eluent was dialyzed 4 times for 10 min with a ‘SUB’ buffer that comprised less Mg^2+^ (20 mM Tris–HCl pH 7.6, 1 mM MgCl_2_, 150 mM KCl, 30 mM NH_4_Cl). The HisTrap^TM^ HP column was then equilibrated with SUB buffer, the sample injected, and the flow-through collected. As the tetra (His)_6_ tag is present only on the 50S subunit, it was trapped in the column whereas the free 30S ribosomal subunit was eluted in the flow-through. The eluted 30S ribosomal subunit was then concentrated using sucrose-cushion centrifugation or 30-kDa cut-off Amicon concentrators (Millipore) to a final concentration of 70 μM, flash frozen and stored in aliquots at −80 °C.

### Sucrose Cushion Centrifugation

The ribosome sample, in a buffer consisting of 20 mM Tris–HCl pH 7.6, 1 mM MgCl_2_, 150 mM KCl, 30 mM NH4Cl, was added to the sucrose cushion solution (1.0 M sucrose, 0.5 M KCl, 0.02 M Tris-HCl pH 7.5, 10 mM MgCl_2_, 0.1 mM EDTA) in a ratio of 2:1. The sample was then centrifuged in thick-walled polycarbonate tubes at 51,000 rpm in a TLA 100.4 rotor on a TLX-120 Beckmann ultracentrifuge for 12 h. The ribosome pellet was then resuspended to obtain the desired concentration^15^.

### Isothermal Titration Calorimetry (ITC)

For ITC experiments, NpmA was first incubated with an excess of SAM/SAH (MP Biomedicals) in a 1:12 molar ratio. Two ml of 30S ribosomal subunit (1.75–2.5 μM) in the sample cell was then titrated with 25–50 μM NpmA-SAM/SAH that was loaded into the injector of a VP-ITC calorimeter (Microcal, LLC) at 20 °C. The samples were degassed and centrifuged before the experiment. The interval between successive injections was 4 min and a volume of 10 μl was dispensed per injection. The ITC data was analyzed using Origin 7.0 (OriginLab Corp.) software.

### Amide Hydrogen/Deuterium (H/D) Exchange Mass Spectrometry (HDXMS)

NpmA was concentrated to 60 μM and the SAH or SAM cofactor was added at a ratio of 1:17 (NpmA: SAH/SAM). Finally, each of the following complexes was used: 60 μM *apo*-NpmA and SAH/SAM-bound NpmA. Deuterium exchange was initiated by dilution of 2 μl of each sample with 18 μl of equivalent buffer reconstituted in D_2_O (pH 7.6) to achieve a final concentration of 90% D_2_O. All samples were kept at 20 °C and the exchange reaction was quenched after 0.5, 1, 2, 5 and 10 min using 40 μl ice-cold buffer containing 0.1% TFA and 2.5 M guanidinium hydrochloride; this addition brought the final pH read of the quenched sample to 2.5. Besides the shorter deuterium labeling times, maximum time of 10 min chosen was suitable as substantial methylation levels have been shown previously at 10-minutes in an *in vitro* methylation assay^16^. This quenched sample (50 μl) was then injected into a chilled nanoUPLC sample manager^17^ and passed at a flow rate of 100 μl/min through a 2.1 × 30-mm immobilized pepsin column (Poroszyme, ABI, Foster City, CA) in a solution of 0.1% (v/v) formic acid in water. The peptides generated by pepsin cleavage were captured by a 2.1 × 5-mm C18 trap (ACQUITY BEH C18 VanGuard pre-column, 1.7 μm resin; Waters). Peptides were eluted using a gradient of 8% to 40% acetonitrile in 0.1% (v/v) formic acid at a flow rate of 40 μl/min and resolved with an ACQUITY UPLC-BEH C18 reversed-phase column (1.0 × 100 mm, 1.7 μm; Waters). The detection and mass measurement of the peptides was performed with a Synapt G2-S_i_ HDXMS mass spectrometer (Waters, Manchester, UK) in the MS^E^ mode, a non-biased, non-selective CID method^18^,^19^,^20^,^21^.

MS^E^ based sequence identification of peptides from non-deuterated samples was carried out with ProteinLynx Global Server 2.4 software (Waters)^21^,^22^ and searched against the sequence of NpmA with a non-modified amino acid and following cleavage by non-specific proteases. The precursor ion mass tolerance was fixed at 10 ppm and fragment ion tolerance at 20 ppm. False discovery rate (FDR) of 4% was set at peptide identification. The peptides that fulfilled these conditions through database search pass 1 were considered. DynamX software was used to calculate the deuterium incorporation in each pepsin-fragment peptide. The instrument was calibrated constantly with Glu-Fibrinogen peptide (GFP) at a concentration of 200 fmol/μL and flow rate of 5 μl/min. The peptides that showed distinct envelopes with favorable signal-to-noise ratios by visual inspection were considered for further analysis. An average of three replicates of deuterium exchange measurements were obtained and are reported in results.

## Results

### Ribosome purification

**Figure 1:**
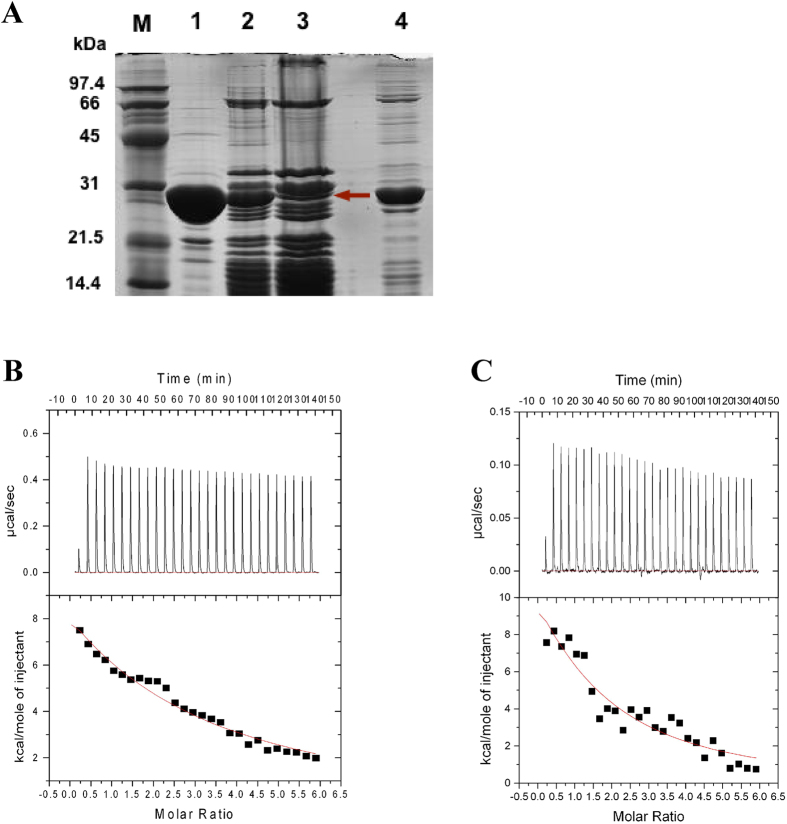
Preliminary binding studies of NpmA with the 30S ribosomal subunit. (**A**) Image of an SDS-PAGE gel depicting the results of sucrose cushion centrifugation performed for the NpmA: 30S complex in the presence of SAH. Lane 1: NpmA-SAH; Lane 2: NpmA-SAH: 30S before sucrose cushion centrifugation; Lane 3: NpmA-SAH: 30S after sucrose cushion centrifugation; and Lane 4: Supernatant after sucrose cushion centrifugation. The red arrow in Lane 3 indicates the NpmA band. (**B**) ITC titration of SAM-bound NpmA into 30S. (**C**) ITC titration of SAH bound NpmA into 30S. In **(B,C)**, the upper panels show the injection profile after baseline correction and the bottom panels show the integration (heat release) for each injection (except the first one).

The 30S ribosomal subunit was purified from an *E. coli* JE28 strain (MG1655 (rplL-his6):kan:rpoB). The eluted 70S (tetra (His)_6_ tagged) ribosome from the nickel affinity column was dialyzed against a buffer containing 1 mM Mg^2+^ to separate the ribosomal subunits (Supplementary Figure 1). Ribosomes obtained by this affinity chromatography method^12^ were found to be of better quality and higher activity when compared with the ribosomes purified by the conventional method^12^. Spedding (1990) demonstrated that the A_260_/A_280_ of the ribosome is indicative of the quality of the intact ribosomal subunit^23^. The SDS gel of the 30S ribosomal subunit (Fig. 1A) showed that all the r-proteins were present in the 30S ribosomal subunit, indicating the good quality of the affinity-purified subunits, and this was consistent with their A_260_/A_280_ ratio of 1.9.

### NpmA-30S ribosomal subunit binding studies

To confirm that NpmA binds to the 30S ribosomal subunit (unmethylated at A1408), sucrose cushion centrifugation was performed with 10–60 μM NpmA and 0.6–1.8 μM 30S ribosomal subunits. Sucrose cushion centrifugation was carried out with *apo*-NpmA:30S, NpmA-SAH:30S and NpmA-SAM:30S samples. The ribosomal pellets obtained were analyzed by SDS-PAGE. An additional band corresponding to the molecular weight of NpmA was observed in the NpmA-SAH: 30S lane (Fig. 1A) but not in the *apo-*NpmA: 30S and NpmA-SAM: 30S samples. This band was confirmed as NpmA by peptide mass fingerprinting (Supplementary Figure 2). These findings suggest that NpmA binds and co-sediments with the unmethylated (at A1408) 30S ribosomal subunit in the SAH-bound form but not in the SAM-bound form, suggesting a transient interaction in the methylated (at A1408) state.

Next, we performed ITC experiments with the SAM/SAH-bound NpmA and the 30S ribosomal subunit. ITC experiments with the ribosome have been previously reported for the mitochondrial ribosome and a mitochondrial inner membrane protein, Oxa1L. Even though the experimental conditions are not the same, the isotherm reported did not reach saturation^24^. Likewise, although the binding isotherm did not saturate in our experiments, the binding affinity (K_d_) for NpmA-SAH:30S and NpmA-SAM:30S was estimated to be 5.1 μM (N = 1.1) and 12.5 μM (N = 0.94), respectively (Fig. 1B,C).

The sucrose cushion centrifugation and ITC experiments both confirmed our previously reported RNA foot printing, methylation assay and computational docking analyses, which showed that NpmA interacts with the purified 30S subunit^9^. We also previously identified potential residues on NpmA important for this interaction: D30, W107 and W197. Therefore, we sought to identify binding sites of SAM/SAH and distinguish it from allosteric sites using HDXMS on NpmA and NpmA-SAM/SAH complexes in solution.

### Amide hydrogen deuterium exchange mass spectrometry (HDXMS) with X-ray crystallography allows identification of SAM/SAH orthosteric and allosteric sites in NpmA

Crystal structures of *apo* NpmA and SAM/SAH bound complexes were reported by us^9^ and others^10^. We identified the residues that form the cofactor-binding site and validated the importance of these residues for cofactor binding, by site-directed mutagenesis and isothermal titration calorimetry experiments^9^. The residues that interact with SAM and SAH were found to be the same although there were differences in the binding affinities of the different variants^9^. First, in order to determine orthosteric and potential allosteric sites of NpmA upon SAM/SAH binding, we carried out amide hydrogen deuterium exchange mass spectrometry (HDXMS) of *apo* and SAM/SAH bound NpmA.

**Figure 2:**
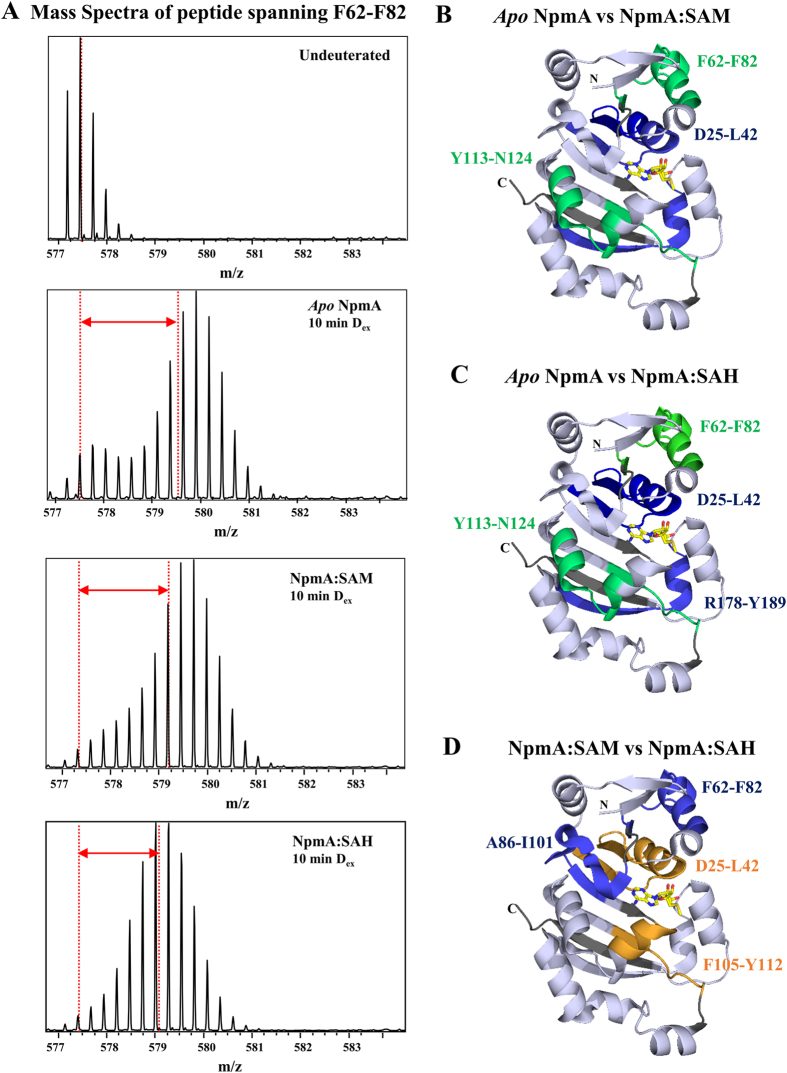
Mapping HDXMS results on the crystal structure of the ligand-bound NpmA (NpmA-SAM, PDB-3MTE, chain B). The results show a decrease in deuteration on binding of NpmA to SAM or SAH. (**A**) Mass spectra for allosteric site spanning peptide F62-F82, for different states of NpmA after 10 min of deuterium labeling time. Top panel shows the isotopic envelope of undeuterated NpmA with the dashed line denoting the mass centroid. Panels below show deuterium exchanged (D_ex_ = 10 min) spectra for apo, SAM and SAH-bound NpmA. Red double headed arrows indicate the shift in centroid with respect to undeuterated state of NpmA. (**B**) Blue colored region indicates the ligand-binding region (D25-L42) and green colored region highlights the allosteric sites (F62-F82, Y113-N124), both showing decrease in deuterium exchange of NpmA:SAM as compared with *apo*-NpmA at 10 min of labeling time. **(C)** Blue colored region indicates the ligand-binding region (D25-L42, R178-Y189) and green colored region highlights the allosteric sites (F62-F82, Y113-N124), both showing decrease in deuterium exchange of NpmA:SAH as compared with *apo*-NpmA after 10 min of labeling time. (**D**) Blue colored region indicates an initial decrease in deuterium exchange of NpmA:SAM as compared with NpmA:SAH, while orange color indicates decrease in deuterium exchange in NpmA:SAH when compared to NpmA:SAM complex at 10 min of deuterium labeling time. Regions with no sequence coverage are in grey. SAM is shown in stick representations.

The sequence coverage of the pepsin proteolyzed NpmA peptides obtained in our HDXMS experiment was 92% (Supplementary Table 1). Examination of deuterium exchange of *apo*-NpmA revealed all peptides spanning residues F62-F82 showed a characteristic bimodal distribution of the isotopic envelope upon deuterium exchange. The isotopic envelope for deuterium exchange (t = 10 min) in the peptide spanning residues F62-F82 is shown in Fig. 2A. This is more prominent at early time points and is clearly seen even after deuterium exchange over 10 min. This is indicative of ensemble behavior of NpmA in solution wherein NpmA populated at least two conformations, one a more ordered conformation and another less ordered conformation in solution^25^. This bimodal distribution is less pronounced in the presence of SAM and furthermore in the presence of SAH.

**Figure 3:**
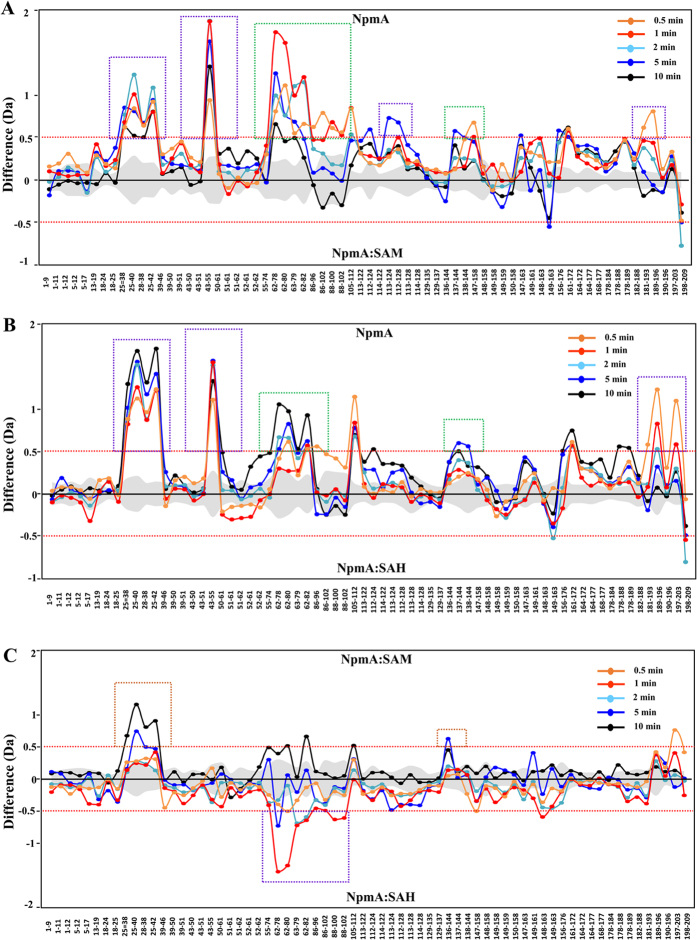
Comparison of amide hydrogen/deuterium exchange for NpmA bound to different ligands. (**A**) Difference plot of the relative deuterium exchange for the pepsin-digested fragments of NpmA in the absence and presence of SAM. Each dot represents a peptide fragment of NpmA and residue numbers are listed from the N- to C-termini on the *x*-axis. The difference (in Daltons) between the two states for each peptide is the difference obtained by subtracting the deuterons exchanged in the top condition (*apo* NpmA) from the deuterons exchanged in the bottom condition (NpmA-SAM complex). A positive value indicates that the peptide exchanged more deuterons (more solvent accessible and/or weaker H-bonding) in the top state than in the bottom state and vice versa for a negative value. Differences in exchange are not corrected for deuterium back exchange in the experiment. Changes in deuterium exchange > ± 0.5 Da (red dashed line) are considered significant for comparative deuterium exchange analysis. Deuterium labeling times of each peptide are depicted according to key. Standard errors are represented in grey. (**B**) Difference plot of the average deuterium exchange for NpmA in the absence and presence of SAH. Details are as described in (**A**). (**C**) Difference plot of the average deuterium exchange for NpmA in complex with SAM (top) or SAH (bottom). Details are as described in (**A**). Boxes in purple highlight the ligand-binding regions (orthosteric sites), while boxes in green highlight the sites of allostery.

A comparison of the deuterium exchange between *apo*-NpmA and the SAM/SAH-bound NpmA complexes showed a decrease in deuterium exchange in peptides in the SAM- (Figs 2B and 3A) and SAH-bound complexes (Figs 2C and 3B). These peptides span regions (i) D25-L42 (peptides D25-N38, D25-Y40, D25-L42, and H28-N38); (ii) A43-D55, (iii) F62-F82 (peptides F62-S78, F62-V80, F62-F82, and D63-N79); (iv) A86-E112 (peptides A86-N96, A86-S102, E88-S100, E88-S102, and F105-E112), (v) A161-L172 and (vi) D181-L196 (peptides V182-E188, D181-F193, Y189-L196 and V190-L196; Supplementary Table 1, Figs 2 and 3). In addition, peptides spanning regions Y113-S144 showed a decrease in deuteration only in the NpmA:SAM complex (Fig. 3A), whereas the peptides R178-E188 and W197-F203 showed decreased deuteration only in the NpmA:SAH complex in comparison to *apo*-NpmA (Supplementary Table 1, Fig. 3B).

The regions D25–D55 and A86–E112 comprise residues that line the SAM/SAH binding pocket and orthosteric site in the corresponding crystal structures and therefore a decrease in deuteration is expected (Supplementary Figure 3)^9^. Interestingly, we observed a decrease in deuteration in regions F62-F82, Y113-S144, A161–E188 and W197-F203, although our previous studies did not implicate these regions in SAM/SAH binding^9^. In the crystal structure of NpmA, we did observe a unique insertion between the β8 and β9 strands corresponding to the D186–F209 region. As W197–F203 is part of this insertion, minor conformational changes that occur in this insertion site, maybe exclusively seen in NpmA and not in other members of the Kam family^9^.

The structures of SAM- and SAH-bound NpmA are identical (except for the side chain orientations of D55 and W107) with the same ligand-binding residues but HDXMS results show an enhanced initial reduction in deuteration in the region F62-F82 in the NpmA-SAM complex as compared with the NpmA-SAH complex (Fig. 2C)^9^,^26^. Due to this difference in deuteration between the SAM- and SAH-bound NpmA, the interaction of 30S and NpmA in the SAM bound state might be preferred. Overall, the decrease in deuteration of F62-F82^26^ reveals that binding of the ligand at the binding site is associated with parallel allosteric, conformational changes at distal sites from the ligand-binding pocket, probably to facilitate ligand-specific binding with the ribosome. This can be attributed to induced conformational changes mediated by ligand-binding^27^ or conformational selection^13^.

## Discussion

X-ray crystallographic studies of NpmA bound to its cofactors, SAH and SAM have provided atomic resolution insights into the cofactor-binding site. HDXMS provides a comprehensive view of conformational changes associated with protein-ligand interactions that encompass both binding and allosteric changes. The HDXMS experiments revealed an overall decrease in deuteration in ligand binding (orthosteric sites) and distal regions of NpmA in the SAM/SAH-bound state compared with its *apo* state. The magnitude of decrease in deuterium exchange was greater in the SAM-bound state than in the SAH-bound state, even though the residues in both SAM and SAH are identical. Interestingly, this decrease in deuteration was also observed in distal regions that do not interact with SAM/SAH, suggesting that ligand binding to NpmA facilitates allosteric effects that are not discerned from the crystal structures.

Importantly, a bimodal distribution of deuterium exchange kinetics was observed in one of the distal regions (F62-F82)^26^, which is indicative of ensemble behavior of NpmA in solution. Of the two conformations, the lower exchanging population possibly represents the 30S subunit-binding conformation. Orthosteric binding of SAM/SAH shifts the conformational equilibrium to favor the more ordered 30S-binding conformation. This mixture of conformations of *apo*-NpmA in solution detected by HDXMS, offers a strong support for Conformational Selection^28^ in explaining SAM/SAH-mediated allostery.

Our foot printing and computational docking studies further suggested a base-flipping mechanism for the A1408 nucleotide of 16S rRNA to facilitate its methylation^9^. From our NpmA crystal structures and using HDXMS to separate changes due to binding^9^,^14^, we have found that F62-F82 is a unique site that is distal to the cofactor-binding site. This region shows decreased deuterium exchange on cofactor-binding and highlights an allosteric hotspot with potential ribosome binding. Moreover, this region has been shown to be important for the initial docking of NpmA onto the 30S ribosomal subunit^29^. Based on our brief NpmA-30S HDXMS experiment^26^ and previous HDXMS studies^14^, we suggest that F62-F82 represents a ribosome interaction site and SAM/SAH allosteric hotspot. Thus cofactor interactions are allosterically coupled to the ribosome binding sites and correspondingly, ribosome binding would allosterically modulate cofactor interactions. Localization of the ribosome-binding site on NpmA within the F62-F82 locus is currently under further examination.

## Additional Information

**How to cite this article**: Husain, N. *et al*. Ligand-mediated changes in conformational dynamics of NpmA: implications for ribosomal interactions. *Sci. Rep.* **6**, 37061; doi: 10.1038/srep37061 (2016).

**Publisher’s note:** Springer Nature remains neutral with regard to jurisdictional claims in published maps and institutional affiliations.

## Supplementary Material

Supplementary Information

## References

[b1] Gonzalez L. S. 3rd & Spencer J. P. Aminoglycosides: a practical review. Am Fam Physician 58, 1811–1820 (1998). 9835856

[b2] Pfister P., Hobbie S., Vicens Q., Bottger E. C. & Westhof E. The molecular basis for A-site mutations conferring aminoglycoside resistance: relationship between ribosomal susceptibility and X-ray crystal structures. Chembiochem 4, 1078–1088, doi: 10.1002/cbic.200300657 (2003). 14523926

[b3] Vicens Q. & Westhof E. Crystal structure of paromomycin docked into the eubacterial ribosomal decoding A site. Structure 9, 647–658 (2001). 11587639 10.1016/s0969-2126(01)00629-3

[b4] Francois B. . Crystal structures of complexes between aminoglycosides and decoding A site oligonucleotides: role of the number of rings and positive charges in the specific binding leading to miscoding. Nucleic acids research 33, 5677–5690, doi: 10.1093/nar/gki862 (2005). 16214802 PMC1251667

[b5] Carter A. P. . Functional insights from the structure of the 30S ribosomal subunit and its interactions with antibiotics. Nature 407, 340–348, doi: 10.1038/35030019 (2000). 11014183

[b6] Brodersen D. E. . The structural basis for the action of the antibiotics tetracycline, pactamycin, and hygromycin B on the 30S ribosomal subunit. Cell 103, 1143–1154 (2000). 11163189 10.1016/s0092-8674(00)00216-6

[b7] Savic M., Lovric J., Tomic T. I., Vasiljevic B. & Conn G. L. Determination of the target nucleosides for members of two families of 16S rRNA methyltransferases that confer resistance to partially overlapping groups of aminoglycoside antibiotics. Nucleic acids research 37, 5420–5431, doi: 10.1093/nar/gkp575 (2009). 19589804 PMC2760815

[b8] Wachino J. . Novel plasmid-mediated 16S rRNA m1A1408 methyltransferase, NpmA, found in a clinically isolated Escherichia coli strain resistant to structurally diverse aminoglycosides. Antimicrobial agents and chemotherapy 51, 4401–4409, doi: 10.1128/AAC.00926-07 (2007). 17875999 PMC2168023

[b9] Husain N. . Structural basis for the methylation of A1408 in 16S rRNA by a panaminoglycoside resistance methyltransferase NpmA from a clinical isolate and analysis of the NpmA interactions with the 30S ribosomal subunit. Nucleic acids research 39, 1903–1918, doi: 10.1093/nar/gkq1033 (2011). 21062819 PMC3061052

[b10] Macmaster R., Zelinskaya N., Savic M., Rankin C. R. & Conn G. L. Structural insights into the function of aminoglycoside-resistance A1408 16S rRNA methyltransferases from antibiotic-producing and human pathogenic bacteria. Nucleic acids research 38, 7791–7799, doi: 10.1093/nar/gkq627 (2010). 20639535 PMC2995053

[b11] Zelinskaya N., Rankin C. R., Macmaster R., Savic M. & Conn G. L. Expression, purification and crystallization of adenosine 1408 aminoglycoside-resistance rRNA methyltransferases for structural studies. Protein expression and purification 75, 89–94, doi: 10.1016/j.pep.2010.07.005 (2011). 20667473 PMC2966526

[b12] Ederth J., Mandava C. S., Dasgupta S. & Sanyal S. A single-step method for purification of active His-tagged ribosomes from a genetically engineered Escherichia coli. Nucleic acids research 37, e15, doi: 10.1093/nar/gkn992 (2009). 19074194 PMC2632923

[b13] Badireddy S. . Cyclic AMP analog blocks kinase activation by stabilizing inactive conformation: conformational selection highlights a new concept in allosteric inhibitor design. Molecular & cellular proteomics: MCP 10, M110 004390, doi: 10.1074/mcp.M110.004390 (2011). PMC3047156 21081668

[b14] Chandramohan A. . Predicting Allosteric Effects from Orthosteric Binding in Hsp90-Ligand Interactions: Implications for Fragment-Based Drug Design. PLoS computational biology 12, e1004840, doi: 10.1371/journal.pcbi.1004840 (2016). 27253209 PMC4890749

[b15] Huber D. . SecA interacts with ribosomes in order to facilitate posttranslational translocation in bacteria. Mol Cell 41, 343–353, doi: 10.1016/j.molcel.2010.12.028 (2011). 21292166

[b16] Savic M. . 30S Subunit-dependent activation of the Sorangium cellulosum So ce56 aminoglycoside resistance-conferring 16S rRNA methyltransferase Kmr. Antimicrobial agents and chemotherapy 59, 2807–2816, doi: 10.1128/AAC.00056-15 (2015). 25733511 PMC4394793

[b17] Wales T. E., Fadgen K. E., Gerhardt G. C. & Engen J. R. High-speed and high-resolution UPLC separation at zero degrees Celsius. Anal Chem 80, 6815–6820, doi: 10.1021/ac8008862 (2008). 18672890 PMC2562353

[b18] Bateman R. H. . A novel precursor ion discovery method on a hybrid quadrupole orthogonal acceleration time-of-flight (Q-TOF) mass spectrometer for studying protein phosphorylation. J Am Soc Mass Spectrom 13, 792–803, doi: 10.1016/S1044-0305(02)00420-8 (2002). 12148804

[b19] Silva J. C. . Quantitative proteomic analysis by accurate mass retention time pairs. Anal Chem 77, 2187–2200, doi: 10.1021/ac048455k (2005). 15801753

[b20] Shen Z. . Label-free quantitative proteomics analysis of etiolated maize seedling leaves during greening. Mol Cell Proteomics 8, 2443–2460, doi: 10.1074/mcp.M900187-MCP200 (2009). 19666873 PMC2773713

[b21] Li G. Z. . Database searching and accounting of multiplexed precursor and product ion spectra from the data independent analysis of simple and complex peptide mixtures. Proteomics 9, 1696–1719, doi: 10.1002/pmic.200800564 (2009). 19294629

[b22] Geromanos S. J. . The detection, correlation, and comparison of peptide precursor and product ions from data independent LC-MS with data dependant LC-MS/MS. Proteomics 9, 1683–1695, doi: 10.1002/pmic.200800562 (2009). 19294628

[b23] Spedding G. Ribosomes and Protein Synthesis: A Practical Approach. (IRL Press, 1990).

[b24] Haque M. E. . Properties of the C-terminal tail of human mitochondrial inner membrane protein Oxa1L and its interactions with mammalian mitochondrial ribosomes. The Journal of biological chemistry 285, 28353–28362, doi: 10.1074/jbc.M110.148262 (2010). 20601428 PMC2934699

[b25] Wang L. C., Morgan L. K., Godakumbura P., Kenney L. J. & Anand G. S. The inner membrane histidine kinase EnvZ senses osmolality via helix-coil transitions in the cytoplasm. EMBO J 31, 2648–2659, doi: 10.1038/emboj.2012.99 (2012). 22543870 PMC3365433

[b26] Husain N. Structural And Functional Studies of Methyltransferases Sgm and Npma that Confer Antibiotic Resistance and their Interactions with the 30s Ribosomal Subunit, Ph.D. Thesis, National University of Singapore (2011).

[b27] Anand G. S. . Cyclic AMP- and (Rp)-cAMPS-induced conformational changes in a complex of the catalytic and regulatory (RI{alpha}) subunits of cyclic AMP-dependent protein kinase. Molecular & cellular proteomics: MCP 9, 2225–2237, doi: 10.1074/mcp.M900388-MCP200 (2010). 20167947 PMC2957725

[b28] Badireddy S. . Cyclic AMP Analog Blocks Kinase Activation by Stabilizing Inactive Conformation: Conformational Selection Highlights a New Concept in Allosteric Inhibitor Design. Molecular & Cellular Proteomics 10, doi: 10.1074/mcp.M110.004390 (2011). PMC3047156 21081668

[b29] Dunkle J. A. . Molecular recognition and modification of the 30S ribosome by the aminoglycoside-resistance methyltransferase NpmA. Proceedings of the National Academy of Sciences of the United States of America, doi: 10.1073/pnas.1402789111 (2014). PMC4035980 24717845

