# Supplementary material for: Ligand-mediated changes in conformational dynamics of NpmA: implications for ribosomal interactions
Source: Sci Rep. 2016 Nov 15;6:37061. doi: 10.1038/srep37061 (PMC5109232; doi:10.1038/srep37061)
Supplement: Supplementary Information [file srep37061-s1.pdf]

Ligand-mediated changes in conformational dynamics of NpmA: implications for ribosomal interactions

Nilofer Husain[1], Nikhil Kumar Tulsian[1], Wang Loo Chien[1], Sushant Suresh[2], Ganesh Srinivasan Anand[1]*, J. Sivaraman[1]*

[1]Department of Biological Sciences, 14 Science Drive 4, National University of Singapore, Singapore 117543

[2]Manipal College of Pharmaceutical Sciences, Manipal, India 576104

SUPPLEMENTARY DATA

Figure Legends

Supplementary Figure 1. Purification of the 30S ribosomal subunit. (A) Affinity purification profile of the tetra $(His)_6$-tagged 70S ribosome purified from the *E.coli* strain, JE28. **(B)** Affinity purification profile of the ribosomal subunits. The 30S ribosomal subunit that eluted in the flow-through was separated from the tetra $(His)_6$-tagged 50S ribosome as it was trapped by the affinity column.

Supplementary Figure 2. Peptide mass fingerprinting. *Top*: The first match (gi|157734594) obtained for the band corresponding to NpmA on the gel is shown. The probability based MOWSE score is 370, which implies that this match is significant. *Below:* The figure depicts the sequence of the match, which is same as that of NpmA. The coverage of the peptides on the protein is also depicted in red.

Supplementary Figure 3. Superposition of the ligand-binding site of NpmA–SAM (AdoMet) and NpmA–SAH (AdoHcy) complexes. AdoHcy is shown in brown and AdoMet in cyan. The residues from the NpmA-SAM and NpmA-SAH

complex are shown in purple and yellow, respectively. PDB coordinates 3P2K and 3P2E were used for NpmA-SAM and NpmA-SAH, respectively.

Supplementary Table 1

Deuterium uptake values for NpmA after 10 min of deuterium exchange in apo NpmA, NpmA:SAH and NpmA:SAM states.

A

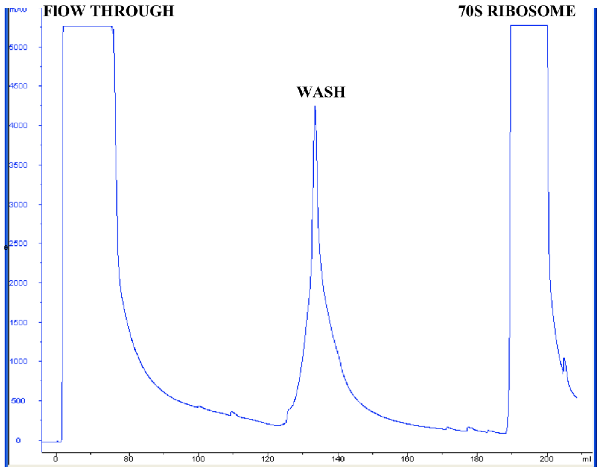

B

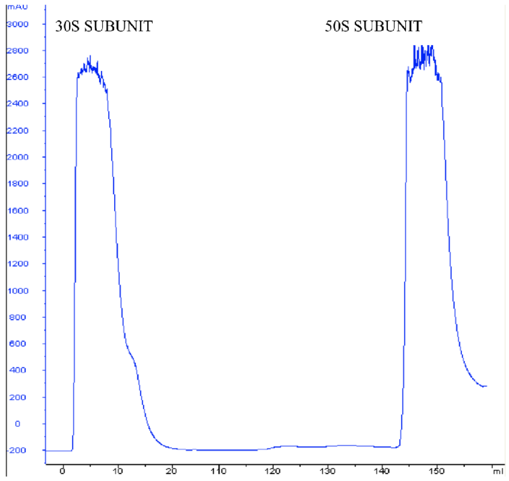

Supp Fig. 1

Probability Based Mowse Score

Ions score is -10*Log(P), where P is the probability that the observed match is a random event.
Individual ions scores > 53 indicate identity or extensive homology (p<0.05).
Protein scores are derived from ions scores as a non-probabilistic basis for ranking protein hits.

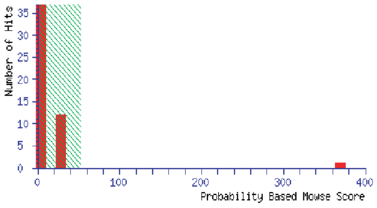

Peptide Summary Report

Format As	Peptide Summary		Help
	Significance threshold p< 0.05	Max. number of hits 20	
	Standard scoring ◉ MudPIT scoring ○	Ions score cut-off 0	Show sub-sets ☐
	Show pop-ups ◉ Suppress pop-ups ○	Sort unassigned Decreasing Score	Require bold red ☐

Select All | Select None | Search Selected | ☐ **Error tolerant** | Archive Report

```
1.     gi|157734594      Mass: 24871    Score: 370     Queries matched: 3
       16S rRNA methylase [Escherichia coli]
```

{MATRIX SCIENCE} Mascot Search Results

Protein View

```
Match to: gi|157734594 Score: 370
16S rRNA methylase [Escherichia coli]

Nominal mass (Mr): 24871; Calculated pI value: 8.54
NCBI BLAST search of gi|157734594 against nr
Unformatted sequence string for pasting into other applications

Taxonomy: Escherichia coli

Fixed modifications: Carbamidomethyl (C)
Variable modifications: Oxidation (M)
Cleavage by Trypsin: cuts C-term side of KR unless next residue is P
Sequence Coverage: 28%

Matched peptides shown in Bold Red

     1 MLILKGTKTV DLSKDELTEI IGQFDRVHID LGTGDGRNIY KLAINDQNTF
    51 YIGIDPVKEN LFDISKKIIK KPSKGGLSNV VFVIAAAESL PFELKNIADS
   101 ISILFPWGTL LEYVIKPNRD ILSNVADLAK KEAHFEFVTT YSDSYEEAEI
   151 KKRGLPLLSK AYFLSEQYKA ELSNSGFRID DVKELDNEYV KQFNSLWAKR
   201 LAFGRKRSFF RVSGHVSKH
```

Show predicted peptides also

Sort Peptides By ◉ Residue Number ○ Increasing Mass ○ Decreasing Mass

Start - End	Observed	Mr(expt)	Mr(calc)	Delta	Miss	Sequence
9 - 26	2079.03	2078.02	2078.05	-0.03	1	K.TVDLSKDELTEIIGQFDR.V (Ions score 152)
75 - 95	2161.14	2160.13	2160.18	-0.05	0	K.GGLSNVVFVIAAAESLPFELK.N (Ions score 112)
96 - 119	2759.46	2758.45	2758.51	-0.05	0	K.NIADSISILFPWGTLLEYVIKPNR.D (Ions score 106)

Supp Fig. 2

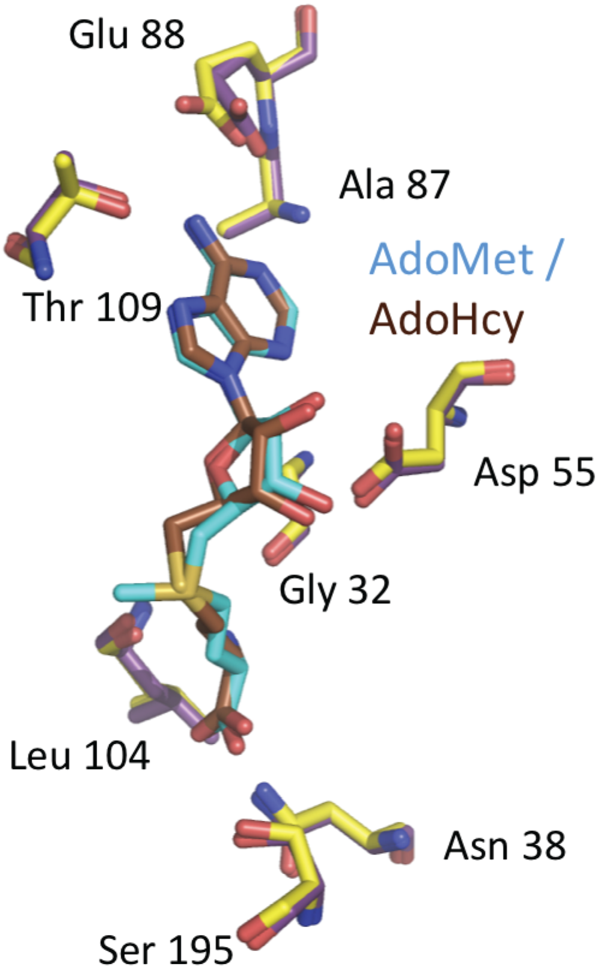

Glu 88
Ala 87
AdoMet /
AdoHcy
Thr 109
Asp 55
Gly 32
Leu 104
Asn 38
Ser 195

Supp Fig. 3

Supplementary Table1: Deuterium uptake values for NpmA after 10 min of deuterium exchange in apo NpmA, NpmA:SAH and NpmA:SAM states.

S.No.	Sequence (MH+)	Residues	z[a]	MEA[b]	NpmA Mean ± SD	NpmA:SAH Mean ± SD	NpmA:SAM Mean ± SD
1	MLILKGTKT (1004.6)	1-9	1	8	2.19 ± 0.04	2.21 ± 0.08	2.3 ± 0.04
2	MLILKGTKTVD (1218.71)	1-10	2	10	2.73 ± 0.04	2.68 ± 0.09	2.79 ± 0.03
3	MLILKGTKTVDL (1331.9)	1-11	2	11	2.72 ± 0.04	2.64 ± 0.1	2.74 ± 0.02
4	KGTKTVDL (861.5)	5-12	2	7	1.83 ± 0.01	1.81 ± 0.06	1.86 ± 0.01
5	KGTKTVDLSKDEL (1433.79)	5-17	2	12	3.17 ± 0.08	3.11 ± 0.1	3.21 ± 0.08
6	SKDELTE (821.39)	13-19	2	6	1.65 ± 0.02	1.61 ± 0.07	1.69 ± 0.04
7	TEIIGQF (807.42)	18-24	2	6	0.89 ± 0.04	0.86 ± 0.02	0.81 ± 0.02
8	TEIIGQFD (922.45)	18-25	1	7	2.03 ± 0.02	1.92 ± 0.03	2.06 ± 0.04
9	DRVHIDLGTGDGRN (1524.75)	25-38	3	13	2.17 ± 0.07	0.87 ± 0.06	1.55 ± 0.13
10	DRVHIDLGTGDGRNIY (1800.9)	25-40	2	15	2.77 ± 0.07	1.09 ± 0.09	2.25 ± 0.1
11	DRVHIDLGTGDGRNIYKL (2042.07)	25-42	4	17	3.06 ± 0.08	1.35 ± 0.07	2.25 ± 0.1
12	HIDLGTGDGRN (1154.55)	28-38	2	10	2.15 ± 0.09	0.84 ± 0.05	1.65 ± 0.1
13	IYKLAIND (949.54)	39-46	2	7	1.64 ± 0.03	1.58 ± 0.04	1.56 ± 0.07
14	IYKLAINDQNTF (1439.75)	39-50	2	11	2.52 ± 0.04	2.31 ± 0.02	2.42 ± 0.05
15	IYKLAINDQNTFY (1602.82)	39-51	2	12	2.39 ± 0.05	2.29 ± 0.08	2.25 ± 0.02
16	AINDQNTF (922.43)	43-50	1	7	2.26 ± 0.03	2.24 ± 0.05	2.31 ± 0.01
17	AINDQNTFY (1085.5)	43-51	2	8	1.95 ± 0.01	1.88 ± 0.04	1.96 ± 0.03
18	AINDQNTFYIGID (1483.7)	43-55	2	12	3.61 ± 0.2	2.28 ± 0.14	2.27 ± 0.16
19	FYIGIDPVKENL (1407.75)	50-61	2	10	2.44 ± 0.04	1.95 ± 0.1	2.16 ± 0.05
20	YIGIDPVKENL (1260.68)	51-61	2	9	2.30 ± 0.01	2.22 ± 0.01	1.93 ± 0.04
21	YIGIDPVKENLF (1407.75)	51-62	2	10	2.07 ± 0.07	2.02 ± 0.1	1.87 ± 0.07
22	IGIDPVKENL (1097.62)	52-61	2	8	2.24 ± 0.04	1.92 ± 0.09	1.91 ± 0.08
23	IGIDPVKENLF (1244.69)	52-62	2	9	2.39 ± 0.04	1.95 ± 0.08	2.13 ± 0.07
24	DPVKENLFDISKKIIKKPSK (2327.37)	55-74	3	17	6.67 ± 0.07	6.19 ± 0.1	6.68 ± 0.18
25	FDISKKIIKKPSKGGLS (1846.12)	62-78	2	15	7.23 ± 0.19	6.17 ± 0.10	6.57 ± 0.06
26	FDISKKIIKKPSKGGLSNV (2059.23)	62-80	2	17	7.35 ± 0.02	6.37 ± 0.18	6.89 ± 0.06
27	FDISKKIIKKPSKGGLSNVVF (2305.36)	62-82	4	19	7.31 ± 0.01	6.38 ± 0.18	7.04 ± 0.09
28	DISKKIIKKPSKGGLSN (1813.09)	63-79	4	15	5.51 ± 0.1	4.97 ± 0.1	5.01 ± 0.13
29	AAESLPFELKN (1218.64)	86-96	2	9	3.77 ± 0.03	3.71 ± 0.03	3.83 ± 0.03
30	AAESLPFELKNIADSIS (1804.93)	86-102	2	15	3.57 ± 0.15	3.82 ± 0.14	3.90 ± 0.14
31	ESLPFELKNIADS (1462.74)	88-100	2	11	2.64 ± 0.23	2.78 ± 0.09	2.80 ± 0.08
32	ESLPFELKNIADSIS (1662.86)	88-102	2	13	2.56 ± 0.21	2.81 ± 0.09	2.86 ± 0.04
33	FPWGTLLE (962.49)	105-112	1	6	3.05 ± 0.01	2.36 ± 0.06	2.88 ± 0.01
34	EYVIKPNRDILSN (1560.84)	112-124	2	11	3.22 ± 0.08	2.69 ± 0.09	2.79 ± 0.05
35	EYVIKPNRDILSNVADL (1959.06)	112-128	3	15	3.15 ± 0.08	2.81 ± 0.12	2.74 ± 0.08

36	YVIKPNRDIL (1230.72)	113-122	3	8	2.23 ± 0.05	1.85 ± 0.06	1.85 ± 0.06
37	YVIKPNRDILSN (1431.79)	113-124	3	10	2.98 ± 0.06	2.63 ± 0.07	2.66 ± 0.04
38	YVIKPNRDILSNVADL (1830.01)	113-128	2	14	2.62 ± 0.1	2.43 ± 0.19	2.49 ± 0.03
39	VIKPNRDIL (1067.66)	114-122	2	7	2.20 ± 0.03	1.85 ± 0.01	1.96 ± 0.02
40	VIKPNRDILSNVADL (1666.95)	114-128	3	13	2.84 ± 0.04	2.75 ± 0.15	2.70 ± 0.06
41	AKKEAHF (830.45)	129-135	2	6	1.25 ± 0.02	1.26 ± 0.01	1.21 ± 0.03
42	AKKEAHFEF (1106.56)	129-137	2	8	1.11 ± 0.01	1.17 ± 0.01	1.18 ± 0.05
43	EFVTTYSDS (1048.45)	136-144	1	8	2.77 ± 0.02	2.39 ± 0.01	2.85 ± 0.04
44	FVTTYSDS (919.40)	137-144	1	7	3.12 ± 0.03	2.62 ± 0.14	2.71 ± 0.06
45	VTTYSDS (772.34)	138-144	1	6	2.82 ± 0.02	2.49 ± 0.21	2.68 ± 0.07
46	EAEIKKRGLPLL (1366.84)	147-158	3	10	5.17 ± 0.05	4.86 ± 0.03	4.95 ± 0.06
47	EAEIKKRGLPLLSKAYF (1963.14)	147-163	3	15	8.28 ± 0.02	7.90 ± 0.09	7.88 ± 0.13
48	AEIKKRGLPLL (1237.8)	148-158	2	9	4.88 ± 0.03	4.68 ± 0.07	4.88 ± 0.08
49	AEIKKRGLPLLSKAYF (1963.14)	148-163	2	14	7.67 ± 0.02	7.67 ± 0.09	7.79 ± 0.1
50	EIKKRGLPLL (1166.76)	149-158	2	8	4.02 ± 0.03	4.11 ± 0.09	4.13 ± 0.06
51	EIKKRGLPLLS (1253.79)	149-159	2	9	4.75 ± 0.02	4.84 ± 0.05	4.94 ± 0.07
52	EIKKRGLPLLSKA (1452.93)	149-161	3	11	5.87 ± 0.05	5.61 ± 0.05	5.72 ± 0.12
53	EIKKRGLPLLSKAYF (1763.06)	149-163	2	13	6.73 ± 0.03	6.96 ± 0.03	7.17 ± 0.07
54	IKKRGLPLL (1037.72)	150-158	2	7	3.33 ± 0.03	3.38 ± 0.06	3.49 ± 0.08
55	PLLSKAYFLSEQYKAELSNSG (2345.20)	156-176	2	19	4.35 ± 0.01	3.84 ± 0.20	3.89 ± 0.15
56	AYFLSEQYKAEL (1461.72)	161-172	2	11	2.86 ± 0.03	2.12 ± 0.05	2.25 ± 0.09
57	LSEQYKAEL (1080.56)	164-172	2	8	2.94 ± 0.02	2.56 ± 0.04	2.64 ± 0.03
58	LSEQYKAELSNSGF (1572.75)	164-177	2	13	4.65 ± 0.03	4.29 ± 0.02	4.29 ± 0.02
59	YKAELSNSGF (1115.54)	168-177	1	9	2.62 ± 0.06	2.22 ± 0.06	2.33 ± 0.03
60	RIDDVKE (874.46)	178-184	2	6	1.62 ± 0.04	1.42 ± 0.04	1.41 ± 0.03
61	RIDDVKELDNE (1345.66)	178-188	2	10	3.42 ± 0.04	2.87 ± 0.04	3.09 ± 0.09
62	RIDDVKELDNEY (1508.72)	178-189	2	11	4.06 ± 0.05	3.52 ± 0.06	3.61 ± 0.03
63	DVKELDNEYVKQF (1626.80)	181-193	2	12	3.58 ± 0.03	3.66 ± 0.09	3.77 ± 0.04
64	VKELDNE (846.42)	182-188	2	6	2.18 ± 0.06	1.96 ± 0.12	2.04 ± 0.06
65	YVKQFNSL (998.53)	189-196	2	7	3.84 ± 0.01	3.77 ± 0.06	3.96 ± 0.04
66	VKQFNSL (835.46)	190-196	1	6	3.12 ± 0.05	3.14 ± 0.05	3.26 ± 0.05
67	WAKRLAF (891.52)	197-203	1	6	3.71 ± 0.01	3.44 ± 0.04	3.58 ± 0.02
68	AKRLAFGRKRSF (1436.86)	198-209	3	11	3.94 ± 0.01	4.32 ± 0.07	4.33 ± 0.09
